# Development of Three-Dimensional Anatomical Models of Dogs with Congenital Extrahepatic Portosystemic Shunts

**DOI:** 10.3390/ani15030352

**Published:** 2025-01-26

**Authors:** Éverton Oliveira Calixto, Erika Toledo da Fonseca, Anna Luiza Campos Pollon, Antônio Chaves de Assís Neto

**Affiliations:** 1 Department of Surgery, School of Veterinary Medicine and Animal Science, University of São Paulo, São Paulo 05508-270, Brazil; evertonoc@usp.br (É.O.C.); erika.fonseca@academico.ufpb.br (E.T.d.F.); annalu.alcp@gmail.com (A.L.C.P.); 2 Department of Veterinary Sciences, Federal University of Paraíba, São Paulo 58051-900, Brazil

**Keywords:** additive manufacturing, shunt, anatomical model

## Abstract

The aim of this study was to develop three-dimensional anatomical models of dogs with congenital extrahepatic portosystemic shunts (CEPSs) using 3D printing, as well as to detail their development process and compare the final models to volume rendering (VR) derived from computed tomography (CT) scans. CT scans in the Digital Imaging and Communications in Medicine (DICOM) format of two canine patients were used—one with splenocaval deviation and the other with right gastrocaval deviation. The images were segmented using 3DSlicer software, generating 3D files in Standard Tessellation Language (STL) format, which were then subjected to refinement and mesh adjustment using Blender software. The models were printed on a J750™ Digital Anatomy™ printer, followed by post-processing in a 2% sodium hydroxide solution for 72 h, with subsequent rinsing to remove support resin residues. The printed models showed colored anatomical structures, including the liver; spleen; kidneys; part of the arterial, venous, and portal circulations; and CEPSs. For comparison purposes, VR of the scans was recreated in the RadiAnt DICOM Viewer software. Despite some limitations of the segmentation software, the 3D-printed models effectively represented the anatomy of the patients and the CEPSs, demonstrating good equivalence to the VR.

## Simple Summary

This study aimed to develop anatomical models of dogs with vascular malformations using 3D printing. This article details their creation process and compares the final models with 3D reconstructions obtained from computed tomography (CT) scans. The CT scans were converted into printable 3D files using 3DSlicer software. These files were then edited using Blender software. The physical anatomical models were generated from the 3D files using the J750™ Digital Anatomy™ printer. The reconstruction of the CT scan for comparison with the 3D models was carried out using the RadiAnt DICOM Viewer software. Despite the limitations observed in the software used, we believe that the anatomical models developed by 3D printing were able to effectively represent the anatomy of the patients and the vascular malformations, demonstrating good equivalence with the 3D reconstructions of the CT scans.

## 1. Introduction

Three-dimensional printing, also known as additive manufacturing, was created in the 1980s with the pioneer development of stereolithography (SLA) by Charles W. Hull [1,2]. Since then, various 3D printing technologies have been introduced, including fused deposition modeling (FDM), selective laser sintering (SLS), digital light processing (DLP), PolyJet, ColorJet, and Inkjet [1,3,4]. These technologies have been widely explored in different areas, including veterinary medicine, since they have great potential for surgical planning, the production of customized prostheses, and interaction with clients and teaching [4]. Three-dimensional anatomical models are highly promising for the surgical planning of complex conditions, such as canine CEPSs [5,6].

These shunts are anomalous vessels that connect the portal system to the systemic venous circulation, diverting blood away from the liver and compromising its essential metabolic functions [7]. CEPSs can originate from veins such as the left gastric, right gastric, or splenic, connecting to the caudal vena cava, phrenic vein, or azygos vein [8,9,10,11,12,13].

Clinically, these deviations result in neurological, gastrointestinal, and urinary signs that significantly impact patients’ quality of life [14].

Initial diagnosis is based on clinical evaluation and serum biochemical tests, such as measuring ammonia and pre- and postprandial bile acids [15]. For definitive diagnosis, advanced imaging tests such as CT or magnetic resonance imaging (MRI) with contrast are used [8,16,17,18]. These methods allow for the precise identification of the anomalous vessel by means of multiplanar reconstructions (MPRs) and VR [19]. Once the CEPSs have been identified, surgical treatment with the placement of ameroid rings or cellophane paper bands is considered the gold standard, since it promotes the gradual closure of the vessel, minimizing the risk of acute portal hypertension [20,21,22]. In this context, detailed surgical planning is crucial for the precise identification of the anomalous vessel and for the success of the intervention.

The objective of this study was to create three-dimensional anatomical models of dogs with CEPSs, to document their entire creation process, and to compare them with VR images, aiming to improve surgical planning and results.

## 2. Materials and Methods

Two contrast CT scans (arterial, portal, and equilibrium phases), in DICOM format, of canine patients with CEPSs were used to develop the models. One patient had a splenocaval deviation and the other had a right gastrocaval deviation. The anatomical structures were segmented using 3DSlicer software version 5.2.1 (GitHub, San Francisco, CA, USA). In this software, six masks were created with the Segment Editor tool and were named as follows: liver, spleen, kidneys, arterial, portal, and venous. The corresponding structures were segmented in each mask, using the contrast phase that provides the best visualization. The kidneys, spleen, liver, aorta, renal artery, celiac artery, cranial mesenteric artery, caudal vena cava, renal veins, portal vein, splenic vein, gastrosplenic vein, gastroduodenal vein, and congenital CEPSs were segmented. The CEPSs were segmented along with the portal circulation. The paint, erase, grow from seeds, margin, and smoothing tools were used in the process. After segmentation, the structures were saved in STL format and exported to a desktop folder using the export/import models and labelmaps tool.

The computer hardware used in this step had the following configurations: Ryzen 5600X processor, Asus TUF Gaming B550M-PlusWIFI II motherboard, RX 6750 XT graphics card, 16 GB DDR4 RAM, 512 GB NVMe SSD, 650w power supply, and Windows 10 64-bit Pro.

The STL files were imported into Blender software, version 3.3.1 (Blender Foundation, Amsterdam, The Netherlands). Surface irregularities and minor modeling on the trigonometric mesh were performed in Sculpt Mode, using the Draw and Smooth tools. At this stage, to improve the appearance of the liver parenchyma, the livers of both models were molded. The position of the spleen was slightly changed with the Move tool (shortcut with the G key on the keyboard) to allow the lateral face of the left kidney to come into contact with the hilar region of the spleen, thus promoting a permanent attachment of this organ to the piece as a whole and eliminating the possibility of the rotation and fracture of the splenic and gastrosplenic veins on the printed model. After refinement, each structure was again exported in STL format.

The J750™ Digital Anatomy™ printer (Stratasys, Eden Prairie, MN, USA) was used to print the models. This printer is connected to a computer with the following hardware: Intel i5-4070 processor, Lenovo SHARKBAY 0b98401 WIN motherboard, Intel (R) HD Graphics 4600 video card, DDDR3 8GB 1600MHz RAM, 120GB Sata lll SSD, 240w power supply, and Windows 10 64-bit Pro.

The STL files were transferred to the computer using an 8GB USB flash drive. On the computer, GrabCAD Print software version 1.62 and 1.74 (Stratasys, Eden Prairie, MN, USA) was used to regulate the printing process. The files were imported to the software using the Add as Assembly tool. Automatic corrections of small defects in the trigonometric mesh were performed with the Model Analysis tool. Automatic adjustment of the model’s position in the print tray was activated with the Arrange tool, in order to optimize time and material. In the Tray Materials tool, Vero family resins were selected for the construction of the models and SUP706 was chosen for the support material. Different colors were assigned to each anatomical structure. The models were printed in High-Speed mode, with a print scale of 120% for the splenocaval deviation and 50% for the right gastrocaval deviation. They were printed separately, and it took approximately 5 h and 19 min, and 1 h and 25 min, respectively.

After printing, the models were immersed in a cleaning vat (Quimis Q215M1) with 2% sodium hydroxide solution for 72 h to remove the support resin. A final rinse in running water was carried out to remove any residue. The volume reconstructions of the CT scans were performed in the RadiAnt DICOM Viewer software (Medixant, Poznan, Poland), versions 2023.1 and 2024.1, using the portal phase of the scans.

## 3. Results

**Figure 1:**
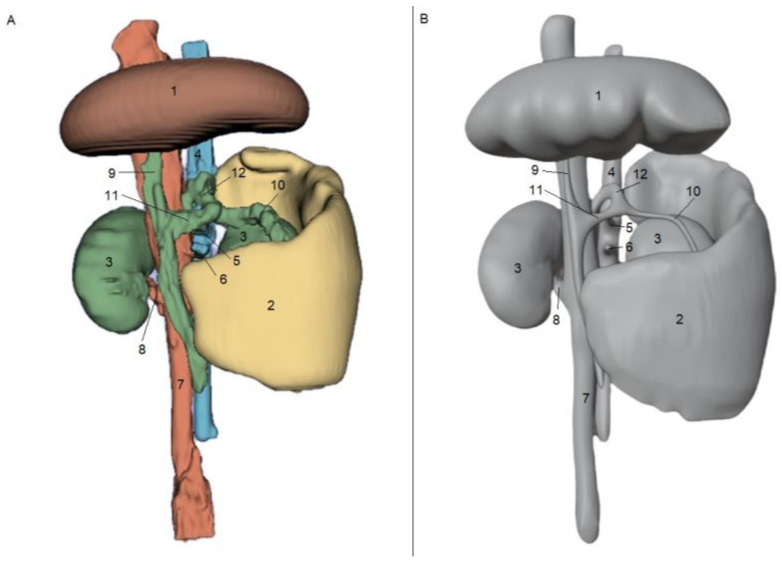
Ventrodorsal view of segmentation obtained from CT scan images in the DICOM format of a patient with a splenocaval shunt in 3DSlicer software (**A**). Image after refinement of the STL file in Blender software (**B**). 1—liver; 2—spleen; 3—kidney; 4—aorta; 5—celiac artery; 6—cranial mesenteric artery; 7—caudal vena cava; 8—renal vein; 9—portal vein; 10—splenic vein; 11—gastrosplenic vein; and 12—splenocaval shunt.

**Figure 2:**
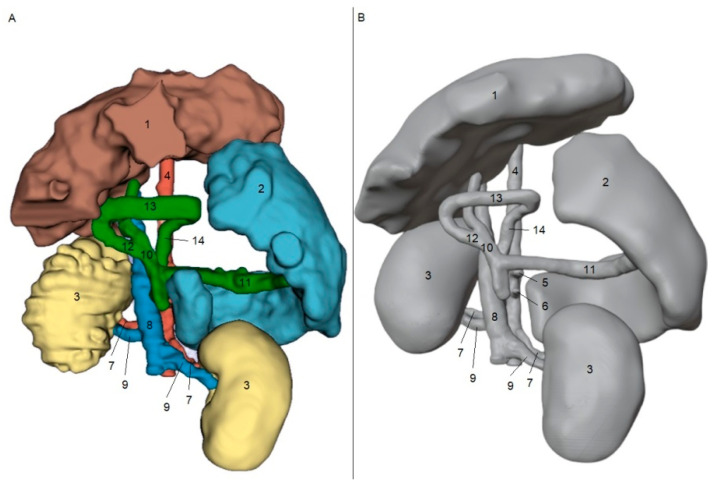
Ventrodorsal view of segmentation from CT scan images of a patient with a right gastrocaval shunt in 3DSlicer software (**A**). Image after refinement of STL file in Blender software (**B**). 1—liver; 2—spleen; 3—kidney; 4—aorta; 5—celiac artery; 6—cranial mesenteric artery; 7—renal artery; 8—caudal vena cava; 9—renal vein; 10—portal vein; 11—splenic vein; 12—gastroduodenal vein; 13—right gastric vein; and 14—right gastrocaval shunt.

Figure 1 and Figure 2 show the results obtained from the processing stages of the three-dimensional models, including initial segmentation in the 3DSlicer software (A) and subsequent refinement in the Blender software (B). Figure 1A and Figure 2A show the initial segmentation using the paint, erase, grow from seeds, margin, and smoothing tools in 3DSlicer. Figure 1B and Figure 2B show the reduction in irregularities in the 3D files, achieved using the draw and smooth tools in the Sculpt mode of the Blender software.

**Figure 3:**
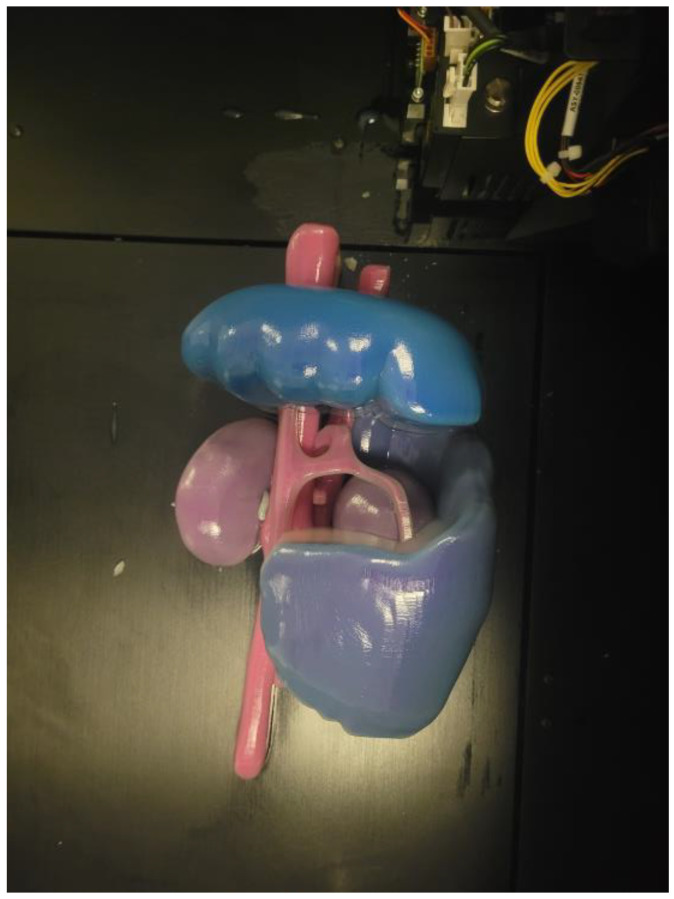
Three-dimensional model of splenocaval shunt immediately after completion of the printing process.

Figure 3 illustrates the three-dimensional model representing a splenocaval CEPS after printing, still covered with the support resin (SUP706).

**Figure 4:**
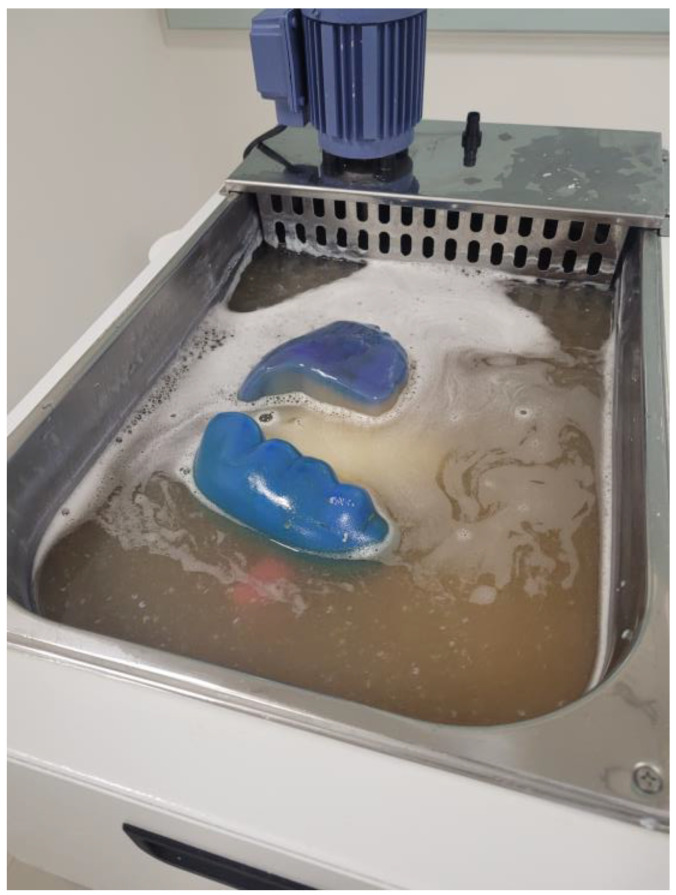
Post processing stage with 2% sodium hydroxide to remove the support resin.

Figure 4 shows the same model immersed in a 2% sodium hydroxide solution, which was used to remove the support resin (SUP706).

**Figure 5:**
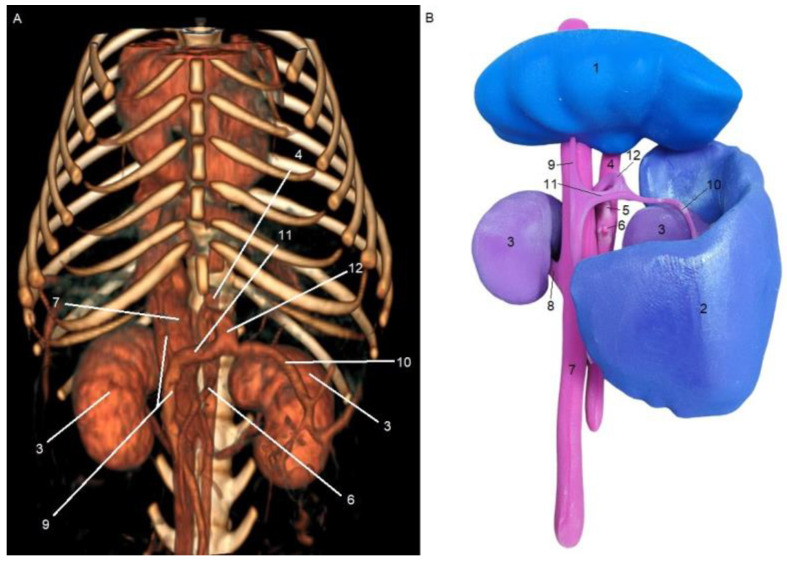
Ventrodorsal view of the volume rendering developed from a DICOM CT scan of a patient with a splenocaval shunt in the RadiAnt DICOM Viewer (**A**). Three-dimensional model produced by the J750™ Digital Anatomy™ 3D printer with a splenocaval shunt (**B**). 1—liver; 2—spleen; 3—kidney; 4—aorta; 5—celiac artery; 6—cranial mesenteric artery; 7—caudal vena cava; 8—renal vein; 9—portal vein; 10—splenic vein; 11—gastrosplenic vein; and 12—splenocaval shunt.

**Figure 6:**
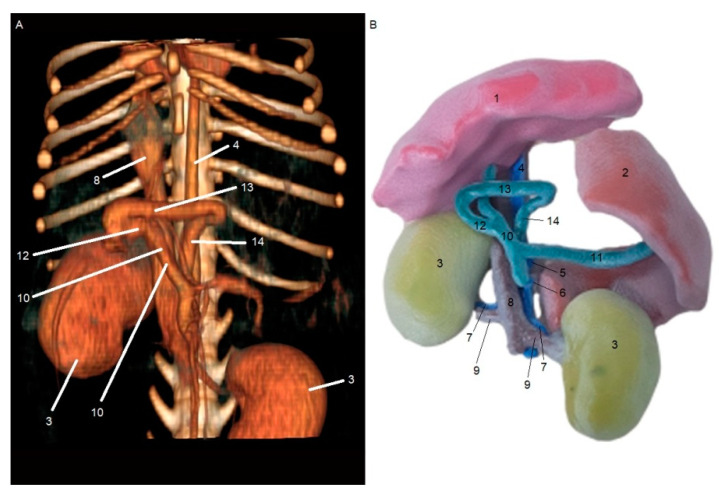
Ventrodorsal view of volume rendering developed from CT scan images of a patient with a right gastrocaval shunt in RadiAnt DICOM Viewer (**A**). Three-dimensional model with a right gastrocaval shunt produced by the J750™ Digital Anatomy™ printer (**B**). 1—liver; 2—spleen; 3—kidney; 4—aorta; 5—celiac artery; 6—cranial mesenteric artery; 7—renal artery; 8—caudal vena cava; 9—renal vein; 10—portal vein; 11—splenic vein; 12—gastroduodenal vein; 13—right gastric vein; and 14—right gastrocaval shunt.

Figure 5 and Figure 6 display the results obtained after VR visualization using RadiAnt DICOM Viewer (A) and the physical model produced by the J750™ Digital Anatomy™ printer after the complete removal of the support resin (B) for the splenocaval and right gastrocaval models, respectively.

## 4. Discussion

The use of 3D-printed anatomical models has shown increasing applicability in clinical and educational practices, especially regarding the surgical planning and understanding of complex pathologies. However, studies often fail to properly describe the development processes of these models, since they usually only list the software used without detailing the stages of development or the specific tools applied. This study sought to fill this gap by thoroughly documenting the development of two canine anatomical models with CEPSs, from segmentation to final printing, and comparing the printed models with the VR images. We consider that in this study, the 3D models were able to represent CEPSs and their topographic relationship with adjacent organs as effectively as VR images, with the advantage of providing a more physical and tactile experience than 3D reconstructions.

In another study that aimed to investigate the normal anatomy of the hepatobiliary circulation in cats using CT, VR, epoxy injection models, and 3D printing, some correspondence was also observed, suggesting that 3D printing can be a valuable tool in the pre-operative planning of hepatobiliary surgeries in that species, including those involving shunts [23].

In canine patients, the 3D printing of anatomical models has already been used to reconstruct congenital intrahepatic and extrahepatic portosystemic shunts and has been shown to help in pre- and trans-operative planning, leading to greater surgical precision, favoring the identification of anomalous vessels and reducing the need for the dissection of the liver parenchyma [5,6]. Three-dimensional printing models with the patient’s individual pathology have already been used in the surgical planning of oral and maxillofacial surgery and also in the pre-operative appointment, helping the patient’s owner to better understand the disease and the procedure that would be performed on their animal [24]. Advanced imaging exams are difficult and complex for clients to understand due to their lack of medical training [25].

CT and VR are widely used tools in the diagnosis, classification, and surgical planning of CEPSs [8,19,26]. In the present study, we identified a deviation originating in the splenic vein and inserted in the pre-hepatic caudal vena cava in one case, and another shunt coming from the right gastric vein, according to the main classifications available for CEPSs. According to Nelson and Nelson’s classification, these types of deviations are called splenocaval and right gastrocaval, while according to White and Parry, they are named left gastrocaval and right gastric of type Ai [8,10,11,27]. Segmentation was carried out using the free 3D Slicer software, which, even though it was effective, had some limitations regarding the individualized segmentation of the liver lobes and the automated segmentation of the blood vessels. Paid software offers a greater variety of tools and user support, and has been widely used in similar studies, such as OsiriX (Pixmeo, Sarl, Geneva, Switzerland), Materialise Mimics (Materialise, Leuven, Belgium), Vitrea (Canon Medical Systems Corporation, Otawara, Japan), Dicom to print (3D Systems Inc., Rock Hill, SC, USA), Avizo (Thermo Fisher Scientific, Waltham, MA, USA), ScanIP (Synopsys, Sunnyvale, CA, USA), AMIRA (Thermo Fisher Scientific, Waltham, MA, USA), Terarecon (Terarecon, Durham, NC, USA), ZedView (LEXI, Chuo-Ku, Tokyo, Japan), M3DICS (M3DICS, Turin, Italy), Volume Extractor (i-Plants Systems, Iwate-ken, Takizawa, Japan), and other non-paid software such as InVesalius (CenPRA, Campinas, São Paulo, Brazil), ITK-SNAP (University of Pennsylvania, Philadelphia, PA, USA), Seg3D (University of Utah, Salt Lake City, UT, USA), and Freesurfer (Massachusetts General Hospital, Boston, MA, USA) [3,28,29,30,31,32,33,34,35]. Commercial software is generally better than free software and provides customer support [36]. As a non-paid alternative, InVesalius was also tested before the beginning of this study; however, it was not selected for use since it showed an inferior performance to 3D Slicer, even on a computer with technical specifications higher than recommended.

During the process, each anatomical structure was segmented into individual masks to enable printing with different kinds of resin, although we opted for rigid resins due to their greater durability. Previous models using flexible materials showed lower resistance. One of our models was produced with a Stratasys PolyJet J850™ printer using resins of different consistencies, such as VeroUltraWhite for bone and Agilus30 for soft tissue, highlighting the versatility of this technology to produce more realistic and functional models [37].

The last step before printing an anatomical model involves using a slicer software, which is responsible for setting up the entire printing process. For Stratasys printers, the compatible software is GrabCAD Print (Stratasys, Eden Prairie, MN, USA). For 3D Systems printers, the most commonly used softwares are 3D Sprint (3D Systems Inc., Rock Hill, SC, USA) and 3DXpert (3D Systems Inc., Rock Hill, SC, USA) [35,38]. For FDM technology, other commonly used free software includes Cura 3D (Ultimaker, Geldermalsen, The Netherlands) and Slic3r (Prusa Research, Prague, Czech Republic), while Simplify3D (Simplify3D, Cincinnati, OH, USA) is a paid software [39,40,41]. Furthermore, ChiTuBox slicer (ChiTu Systems, Shenzhen, China) is widely used with resin printers [42]. It is important to note that each type of printing technology works with specific software; therefore, compatibility must be checked, as there is a wide range of software available.

All types of software (segmentation, 3D editing, or slicing) offer a wide variety of tools and functionalities for model production. Therefore, each of these software programs has a different layout and requires specific learning, which makes teaching the creation of an anatomical model a challenge. It is likely that only a practical course dedicated to each specific software would guarantee proficiency in their functionalities.

In this study, we used a J750™ Digital Anatomy™ printer. The advantages of Polyjet technology are its greater precision, finer finish of the pieces, possibility of printing models with multiple materials and colors, and easy removal of the support resin [43]. Polyjet printers provide more realistic prints and improved educational experiences compared to FDM printers; on the other hand, they are more expensive [1,44]. Even so, models of extrahepatic and intrahepatic portosystemic deviations and feline hepatobiliary circulation have been produced with simpler printers, using FDM and mSLA technologies, which are similar to SLA but perform photopolymerization with an LCD screen [5,6,23]. Therefore, it is also possible to make these models with smaller, cheaper printers and less expensive materials. After the patent break, 3D printers with FDM and SLA technology have become more accessible and relatively cheap [25].

The only treatment performed on the model after printing was immersion in a 2% sodium hydroxide solution to remove the SUP706 support resin; however, the manufacturer’s instructions also state that this can be removed manually or with a high-pressure water jet. PolyJet technology eliminates support artifacts and guarantees a higher quality surface compared to FDM printers [1]. Depending on the type of technology and the support material employed, other treatments may be necessary after printing, such as photopolymerization (UV light), manual finishing (usually for FDM), using acetone vapor (ABS filament in FMD), bringing color to monochrome models (manual painting), and removing any residue from the support material (blasting with glass beads, using instruments, using a high-pressure water jet, or immersing in isopropyl alcohol) [1,5,6,45,46,47,48,49,50,51].

After producing the models and understanding all their development stages, it was possible to see that the final quality of a 3D-printed anatomical model depends directly on several factors, i.e., the quality of the CT scan or MRI images, the ability to interpret these images, anatomical knowledge, the efficiency of the segmentation software, mastery of the software tools, familiarity with 3D modeling, experience with handling 3D printers, and proficiency in the techniques used to finish the 3D-printed pieces.

It took us approximately 60 days to make the first model (splenocaval) and 10 days for the second (right gastrocaval). These were the first 3D-printed models developed by the authors. There was a need to improve our interpretation of CT scan images, as well as a need to learn how to use the software and its most important tools for this task. After the production of the first model, the process of making the second one became easier. Previous studies have reported a production time of 7 to 10 days for models with intrahepatic portosystemic shunts [6]. Those authors were probably already familiar with this process.

A limitation of this study was the use of a free segmentation software, which brought some restrictions and made the process more laborious, and it also required the use of 3D editing programs such as Blender. The refinement and modeling processes employed may generate models that are not one hundred percent faithful to the patients. Moreover, the printing scale was not performed in a 1:1 ratio, since this would make it impossible to use the models in specific surgical simulations of these patients or in the trans-operative period.

Developing three-dimensional anatomical models requires a high degree of expertise in interpreting medical images and handling segmentation and modeling software, as well as experience in using 3D printers and finishing techniques. Albeit challenging, the advancement of this technology provides promising educational and diagnostic tools, expanding access to prototyping in clinical practice.

## 5. Conclusions

The anatomical models developed in this study satisfactorily reproduced the anatomy of CEPSs in dogs, based on VR. Although not completely reliable due to the printing scale and the limitations of the process, the models showed great potential for improving anatomical understanding, surgical planning, and veterinary education. Advances in the technical mastery of segmentation, modeling, and 3D printing processes might enable the creation of more accurate and functional models. Future studies should explore the validation of these models in clinical and educational contexts, as well as investigating ways to optimize the workflow to make it more accessible to healthcare professionals.

## Data Availability

The original contributions presented in this study are included in the article. Further inquiries can be directed to the corresponding author.
